# Anatomical Description of an Hermaphrodite, Known by Turns under the Names of Marie-Dorothú Derier, and Charles Durgé

**Published:** 1841-04-10

**Authors:** 

**Affiliations:** Bon.


*Anatomical description of an Hermaphrodite, known by turns under the names of Marie-Dorothú Derier, and Charles Durgé.* By Professor MAYER, of Bon. Translated from the French, by O. H. PARTRIDGE, M. D., of Philadelphia.

This remarkable individual whom we are about to describe, has been known to the medical community for more than thirty years, at first by the name of Marie-Dorothú Derier, afterwards by that of Charles Durgé. Born at Berlin or Potsdam in 1780; he was baptized as a female child. At twenty years of age he still continued to wear the clothing and follow the occupation of females moving in the same grade of life; it was about this time that he was first spoken of in the public journals. Hufeland made mention of him in his journal for 1801; and notwithstanding the perspicacity of this patriarch in German medicine, he gave it as his opinion that the attributes of the female sex were predominant with Derier.

During the years 1816 and 1817, Derier, who had learned to model in wax, visited the different universities in Germany, and travelled into France, Holland, and England, where he was employed in most of the anatomical museums. He was examined by a great number of medical gentlemen and men of science; among whom were Kopp, Kansch, Marsina, Rosenmüller, Osiander, Lawrence, Green, and the faculty of medicine in Paris, the most of whom gave it as their opinion that he was of the masculine sex. Hufeland, whom we have already mentioned, Gall, and Brooks, declared in favour of the feminine. While others, and among them Drs. Schneider and South, Schmitmüller and Ritgen, were of the opinion that Derier belonged to neither sex; the different parts of the body were considered as appertaining to the masculine and partly to the feminine. The pelvic basin only was considered by the most of them as the basin of the female; notwithstanding, the anatomical inspection, as we shall see, has demonstrated to the contrary.

Supported by the opinions of a majority of physicians, who had pronounced him of the masculine sex, and stimulated by a sort of vanity, Derier assumed the dress and mode of living usual among men, and caused himself to be called Charles Durgé. Under this name he continued to live in Bon, from 1820 to the time of his death, which occurred in March, 1835. During the latter period of his life he was constantly under the observation of M. Mayer. Durgé, said he, was pleased to be in the society of males, but evinced a greater predilection for that of females, yet entertained towards neither of them any amorous propensities. His character was a *mélange* of the man and the woman: on the one part, he had courage unusual to one of his size,—he possessed great physical strength, and loved to rule; and, on the other, distinguished himself for great manual dexterity, as his works in wax were always well and faithfully made. He was animated with sentiments mild and affectionate, yet at the same time possessed a certain spirit for contradiction.

During the last years of his life there was no evidence of a catamenial discharge from the genital organs, as there had been two or three times during his twentieth year, yet he was subject to epistaxis and hæmorrhoids, phenomena which were attributed to his manner of living, as he indulged freely in the use of wine, coffee, and spirituous liquors. Durgé never had pollutions or ejaculations of semen. His voice became more hoarse and strong as he advanced in age; his beard was light and thin; all his hair had fallen off, with the exception of a few locks that hung long and pendant from behind the occiput. His head and face presented the aspect of an old woman; the teeth also were nearly all gone; neck short; chest fat and rotund; arms and legs slightly covered like those of the female; his height, which was taken at the time he was thirteen years of age, when he had arrived at his full stature, was five* feet. At thirty-eight there was a complete change in his constitution, and he became very gross and portly; he enjoyed most excellent health, with the exception of a nervous fever, which attacked him when he was about forty years old. He continued thus to live until three years previous to his death, when his memory failed, and he lost all taste for his works in wax. In the month of March, 1835, his countenance assumed a haggard aspect for a number of days, at the end of which Durgé or Derier succumbed suddenly of a violent attack of apoplexy. The autopsy was made by Professor Mayer, one of the most distinguished anatomists of Germany. M. Mayer was exceedingly minute in his examinations, which might have been thought by some unnecessary, had they not been justified by the importance of the subject.

We have thought it our duty to report faithfully the description of the professor in Bon, because it has thrown much light upon the true nature of an individual who had enjoyed a certain celebrity in consequence of the great difficulty that had existed to determine to which sex he belonged. It proves to us, moreover, that there does exist among the human species, true hermaphrodites,—that is, the organs male and female in the same individual.

*Autopsy Exterior.*—Length of the body 5 feet; length of the superior extremities from the condyle of the humerus to the end of the middle finger 2 feet 4½ inches; inferior extremities from the great trochanter to the heel 2 feet 10¾ inches. Form of the head feminine, small,

* We here make use of the French measure, which the reader will recollect is a little longer than the English—12 French inches being equal to about 13 English inches.—*Tr.*

os frontis, narrow and low; occiput prominent; hair thin, and covering only the occipital region; beard very thin; neck short; larynx slightly projecting; breadth of the shoulders 1 foot 2 inches; superior portion of the thorax narrow and short; abdomen of good length; breasts very well developed, but the nipples shrunk; pelvis not large; pubien arch of a medium width; curve of the arms and legs like those of the female.

*Description of the internal organs.*—Tongue short, large, and ovoid; papilla long and of a natural size; os hyoides small, but well developed; the thyroid cartilage forms but a slight projection, is very narrow, but of a tissue sufficiently hard throughout; thyroid gland quite large; cornua thyroidea, superior and inferior, very long; the ligaments of the thyroid and cricoid cartilages, strong and well developed; epiglottis short and large; cavity of the larynx moderately large, neverthelesss, the vocal chords, superior and inferior, are sufficiently thick and strong; trachea narrow, and its cartilages more soft than those of the male. Lungs small; the right lung is divided only into two lobes by a fissure incomplete and not very deep; the left also is divided and adherent to the parietes of the thorax, the pericardium and diaphragm; the right lung, with the exception of a few arthritic tubercles of the size of peas, are healthy; the left contains a large number of these concretions, particularly on the internal edge of the superior lobe. Heart fat, large, round, and resembles the heart of a female; its structure is normal, and the muscular tissue well nourished; the division of its vessels from the arch of the aorta, natural. The stomach is of an oblong form and not very muscular. Spleen small, and only 2 inches 10 lines in length, and 1 inch 8 lines broad. Liver of a medium size, biliary ducts contain a large number of calculi. Intestinal canal normal; cœcum large; appendicula vermiformis large. Mamma tolerably well developed, the glandular granulations are wanting, but in their place are found a quantity of small globules, fatty, and of a reddish yellow appearance; the nipple projects but slightly, and is pierced by a great number of minute holes, which are evidently sebacious follicles, and situated only about the areola. Kidneys oblong, thin, and small; renal capsules normal.

The encephalon is small, and presents entirely the form and organization of that organ in the female; its form is round and symmetrically convex; lobes not prominent, convolutions numerous, but narrow; dura mater and pia mater are of an extremely delicate texture; pons varoli and medulla oblongata small; the nerves, and particularly the fifth pair, are more delicate than in the male; the cerebellum is also small, and its right hemisphere in particular is much less voluminous; in fact all the lobes appear to have been arrested in their development; the lamina are numerous; the cerebrum presents also a want of development in its right hemisphere, as is indicated by a depression at the lobes; corpus callosum short; thalami nervorum opticorum, corpora quadrigemina, pineal gland and corpora striata are below the natural size. *Cranium* small; its bones thin but solid; sutures not effaced; bones of the face tolerably well developed; ossa maxillaria superiora destitute of teeth; in the ossa maxillaria inferiora two molar and three incisors still remain; mastoid processes large and of a very firm texture; os frontis slightly projecting, but the summit of the cranium and the occiput which contain the posterior lobes are quite large; the prominences corresponding to the cerebellum, not well developed, the left saillant and the right quite smooth; vertebral column regularly formed, but the vertebræ, especially the cervical and thoracic, are of rather delicate texture; ribs brittle and thin; the 3d, 4th, 5th, 6th and 7th on the left side have been fractured, the four first in two places, the last in one only; the fractures now are consolidated and quite firm; the pleura adheres at three different points, and also to the lungs; the sternum, particularly its superior portion, is quite large; thorax uncommonly narrow, more especially its middle portion, while its lower section is also of good size.

The bones of the superior extremities are proportionally well developed, but present a feminine character, particularly the clavicle and scapula; the first is short, rounded, thin, and very curved—the fore-arm forms with the arm an angle from without, a little unusual; the hands are small like those of the female; lumbar vertebra rather small, sacrum large; sacro vertebral angle slightly prominent. The bones of the pelvis are strong and solid, and in general narrow, presenting in an evident manner the configuration of the pelvis of a male; its greatest transverse diameter from the crest of one ilium to the other is 9 inches; from the anterior superior spinous process of one ilium to the opposite it is 7 inches and three lines; transverse diameter of the superior strait 4 inches 5 lines; antero posterior 3 inches 4 lines; oblique diameter 4 inches; transverse diameter of the inferior strait 3 inches 3 lines; antero posterior 2 inches 6 lines; symphisis pelvis long and narrow; the arch of the pelvis is like that of the male, and forms an angle of 65 degrees; the lateral portions of the ilium have a vertical direction; cotyloid cavities are flat and turned forward; sciatic tuberosities look downward and inward; the whole of the pelvis is a little unequal and oblique, inasmuch as the right half of the small basin is smaller and more narrow than that of the left, and the sacro vertebral angle greater towards this side; osseous tissue of the inferior extremities very delicate; neck of the femur short; trochanters feeble; knees a little crooked from within.

*Description of the genital organs in particular.*—Mons veneris slightly elevated; the hair that cover it are thinly scattered and do not extend as far as the umbilicus; perineum and anal region also only partially covered. Length of the penis to the glans two inches; the glans itself nine lines; penis mostly covered by the mons veneris; corpus cavernosum equally well developed, presenting, each one a perpendicular diameter of eight lines, and together a transverse diameter of four lines, divided by a septum; corpus spongiosum wanting; the prepuce covers but about half of the glans; and a little on the under side is found an opening, *fossette naviculaire*, or small canal, which represents the canal of the urethra closed, and is formed of two folds of skin, stretched backwards, and which resemble the nympha; this small canal leads, to an opening, round and about the size of a goose quill; the external labia, the skin of which is wrinkled, forms the posterior edge of this opening, and the mucous tissue, smooth and glossy, its anterior; at the anterior or superior border are to be seen two longitudinal folds of skin, between which is a small canal representing the canal of the urethra, and runs in a direction inward; at the edges of the internal labia are also seen the vestiges of the curunculæ myrtiformis; the circular opening continues, with a vestibule of eight lines in length, and merges above into the canal of the urethra, and below into a larger canal which resembles a vagina; the septum that divides at this place the vaginal and urethral canals is semilunar in form, and is placed horizontally; the canal of the urethra is near the racine of the penis, and is at the same time surrounded by the prostate, which is firm, but not very thick; neck of the bladder, and the bladder itself, are regularly formed; the latter particularly is very muscular, and its membranes dense and strong; the mouths of the ureters present nothing singular. The canal which represents the vagina is composed of a delicate mucous tissue, and has but few muscular fibres, and is partially filled with a greenish mucus; at its commencement it is surrounded by a tissue, rectiform and vascular, and easily separated; this tissue is composed mostly of varicose veins, which continue upwards between the vagina and uterus, and finally disappear; at this place are seen a number of veins coming out, and a large artery entering. Length of the vaginal canal two inches eight lines; diameter of the external orifice, where it is the largest, ten lines; posteriorly, it is only six lines; in its internal surface anteriorly it is a little corrugated,—farther back, smooth, but garnished with a great number of small warts, where are to be seen also radiated or star-formed cicatrices. The vagina terminates interiorly by a narrow portion or sort of isthmus of a spongy texture, and of from four to six lines in length; behind this isthmus, which represents the imperforate orifices of the uterus, is found the uterus itself, which continues upwards with the oblique direction of the vagina, behind and between the bladder and rectum, with a slight declination from right to left, so that its base is seen on the left side of the bladder, at the point of union between the body and base of this organ.

The uterus is extremely narrow; length two inches six lines; neck quite small, yet it is not difficult to distinguish that it has a neck, body, and fundus; there is nothing remarkable in the internal surface, except some small folds and a number of small spots of a yellowish brown colour; its cavity, which contains a gelatinous mucous, is much narrower than that of the vagina, and will hardly receive a goose quill; its fundus is a little larger, and will measure nearly six lines; the body of the uterus exhibits a few corrugations or folds, and a number of hydatiforme vesicles, mixed here and there with yellow spots.

The two Fallopian tubes open exactly at the fundus of the uterus, but in length are unequal; the left tube being three inches four lines, and the right four inches four lines; the tube of the canals is small, but perfectly permeable to the abdominal opening, which is imperforate, and where are discovered a number of hydatids; corpus Fimbriatum plainly to be seen; the muscular fibres are very strong which are given off from the fundus of the uterus, under the peritoneum, and which pass over its anterior portion and the bladder in a direction towards the inguinal ring, and finally are lost in the adipose tissue of the external organs. In the right side, near the open extremity of the Fallopian tube, is a small, flat, ovoid body, from which are given off a bundle of vessels and muscular fibres, and entirely enveloped in the peritoneum; its form is that of a small almond; its parenchyma is evidently composed of a yellow, soft, and filamentous tissue, resembling exactly that of the testicle; the vesiculæ seminales may easily be dissected, and in the cord are to be seen the artery and vein. On the right side, behind and a little without the abdominal opening of the Fallopian tube, there is also a small, round, flat body enveloped in the peritoneum, but its tissue is gelatinous, and composed of small grains, so conglomerated as to resemble rather an ovary than a testicle."

We here see the mixed attributes of a male and female; on the one part a testicle slightly atrophied, also a penis and prostate gland; on the other, a vagina and uterus with its Fallopian tubes, and on the left side a body analagous to an ovary. M. Mayer has also observed as a fact worthy of special notice, the very slight development of the hemispheres of the cerebellum, an anomaly that Gall predicted, when Derier or Durgè was twenty-five years of age, in consequence of the indifference exhibited by him for either sex. It is also a fact, that on the right side, where the cerebrum, and particularly the cerebellum, were imperfectly developed, there exists in the abdominal cavity but one thing doubtful, that is, the body which was supposed to be an ovaria; while on the left side there is certainly a testicle, although not of full size.

Finally, M. Mayer adds, he never has observed this defect in the organization of the cerebellum in any other case of hermaphroditism, either in the male or female.

